# Dietary encapsulated *Bifidobacterium animalis* and Agave fructans improve growth performance, health parameters, and immune response in broiler chickens

**DOI:** 10.5713/ab.21.0213

**Published:** 2021-08-25

**Authors:** María José Hernández-Granados, Rosa Isela Ortiz-Basurto, Maribel Jiménez-Fernández, Carlos Alberto García-Munguía, Elena Franco-Robles

**Affiliations:** 1 Interinstitutional Master’s degree in Livestock Production, Division of Life Sciences, Campus Irapuato-Salamanca, University of Guanajuato, Irapuato, Guanajuato 36500, Mexico; 2 Integral Food Research Laboratory, TecNM-Institute Technological of Tepic, 2595 Technological Av., Tepic, Nayarit, 63175, México; 3 Food Research and Development Center, Veracruzana University, Xalapa, Veracruz 91190, Mexico; 4 Department of Veterinary and Zootechnics, Division of Life Sciences, Campus Irapuato-Salamanca, University of Guanajuato, Irapuato, Guanajuato 36500, Mexico

**Keywords:** Interferon-gamma, Interleukin 10, Leukocytes, Probiotics, Prebiotics, Tumor Necrosis Factor

## Abstract

**Objective:**

The present study was conducted to evaluate the effects of dietary supplementation with *Bifidobacterium animalis*, Agave fructans, and symbiotic of both encapsulated on growth performance, feed efficiency, blood parameters, and immune status in broiler chickens, and to compare these with diets including antibiotic growth promoters and without additives.

**Methods:**

A comparative experimental study was carried out with 135 male Ross 308 broiler chickens. Each trial was divided into 5 equal groups. Control group (CON) received a standard diet without growth promoter; GPA, a standard diet with colistin sulfate and zinc bacitracin (0.25 g/kg of feed); PRE, a standard diet with 1% Agave fructans; PRO, a standard diet with *Bifidobacterium animalis* (11.14±0.70 log CFU/g); SYM, a standard diet with *B. animalis* and Agave fructans.

**Results:**

A significant decrease in food consumption was found for the GPA, PRE, and SYM, compared to the CON group. The results show a better feed conversion index in PRE and GPA with respect to the CON group with the highest conversion index. Interestingly, the weight of the gastrointestinal tract shows a statistically significant difference between GPA and PRE groups. Moreover, the length of the gastrointestinal tract of the GPA group was less than the PRE group. In the total leukocyte count, there was a statistically significant increase in the GPA group compared to the CON, PRE, and PRO groups, and the heterophiles-lymphocytes index was lower in PRO. Regarding the cytokines, interleukin 10 (IL-10) decreased in PRO compared to CON and PRE, while IL-1β increased in the SYM group.

**Conclusion:**

Alternative treatments were shown to achieve similar productive results as growth-promoting antibiotics and showed improvement over diet without additives; however, they have immunomodulatory properties and improved the development of the gastrointestinal tract compared to the treatment of growth-promoting antibiotics.

## INTRODUCTION

In broiler diets, antibiotics are used to improve growth performance and are known as growth-promoting antibiotics (GPAs). Also, they are used as a prophylactic therapy to prevent the development and transmission of diseases [1]. It has been proposed that GPAs suppress the production of catabolic mediators by intestinal inflammatory cells, thus altering the normal microbiota, while others suggest that GPAs induces changes in the microbiota that reduce inflammatory interactions in the small intestine [2]. Further, it has been proposed that after an inflammatory stimulus, such as exposure to infectious pathogens and their toxins, GPAs generate an anti-inflammatory effect by reducing the production of cytokines and chemokines, which leads to a decrease in muscle catabolism and a decrease in anorexia [3]. Moreover, chickens treated with penicillin and streptomycin show a decrease in cytokines related to inflammation in the cecum [4]. Accordingly, despite the wide acceptance of their use for the supposed benefits they confer on poultry, the mechanisms of action remain unknown [5].

Probiotics have been used to improve animal performance and maintain the normal microbiota of host animals. Their main action is a reinforcement of the intestinal mucosa barrier against adverse agents [6]. Prebiotics like fructans are a promising alternative for the poultry industry due to their ability to cross the digestive tract, facilitate, and support the symbiotic relationship between the host and the microbiota of the gastrointestinal tract (GIT), and produce health benefits [7,8]. The benefits of *Bifidobacterium animalis* and Agave fructans are promoted more efficiently when both work together in the living system since the symbiotic relationship contributes significantly to health [1]. Symbiotics stimulate the growth of the probiotic organism by providing the specific substrate to the probiotic organism for fermentation [9]. The aim of this study was to evaluate the effect of a probiotic, prebiotic, and symbiotic encapsulated on growth performance, feed efficiency, hematological and biochemical parameters, small intestine characteristics, as well as cytokines and immunoglobulin A (IgA) in broiler chickens.

## MATERIALS AND METHODS

### Encapsulated treatments

The symbiotic was composed of *Bifidobacterium animalis* subsp. lactis (BLC-1) (11.14±0.70 log CFU/g) and 10 g of native Agave fructans (Mieles Campos Azules, Amatitán, Jalisco, México) in a double emulsion. Development, production, and characterization of probiotic, prebiotic, and symbiotic capsules were performed and described by Juárez-Trujillo et al [10].

### Birds, housing, and diets

The experimental protocol was performed following the guidelines of chapter 7.10 of the Terrestrial Animal Health Code of the World Organization for Animal Health [11] and the management and rearing manuals for animal welfare, always analyzing their behavior and continue taking care of the environment. The research protocol was approved by the Institutional Committee of Bioethics in Research of the University of Guanajuato with the code CIBIUG-P07-2019.

A comparative experimental study was performed with a total of 135 one-day-old male broiler chickens Ross 308 (Aviagen Group, Huntsville, AL, USA), with an average weight of 42.38±3.5 g. The birds were randomly distributed into five groups with 27 birds each. The broilers were housed in 15 experimental units consisting of a pen of 1 m×1 m each. All birds were administered with an initial standard diet (first 3 weeks: crude protein [CP], 23%; metabolizable energy [ME], 3 kcal/kg) and finisher standard diet (the last 4 weeks: CP, 21%; ME, 3.22 kcal/kg) with or without the following additives (Table 1). Treatment 1 (control, CON), animals fed a standard starting and finishing diets, without GPA; treatment 2 (GPA), animals fed a standard starter and final diets with 0.25 g/kg feed of colistin sulfate and zinc bacitracin COLI-ZIN (PRODE International laboratory); treatment 3 (PRE), animals fed with a standard starting and finishing diets with prebiotic capsules with 1% of Agave fructans; treatment 4 (PRO), animals fed a standard starting and finishing diets with probiotic capsules with *Bifidobacterium animalis* (11.14±0.70 log CFU/g); treatment 5 (SYM), animals fed with a standard starting and ending diets with symbiotic capsules (11.14±0.70 log CFU/g+10 g Agave fructans). Food was offered *ad libitum* in poultry hopper feeders and the leftover was weighed each day to obtain the food consumed. Drinking water was also provided *ad libitum* daily in glass-bottom waterers.

The euthanasia of the broiler chickens was performed at the end of the experiment (49 days) using the cervical dislocation technique described in the “Manual for the welfare of broiler chickens” published by the National Service of Health, Safety and Agri-Food Quality of Mexico [12] and NOM-033-SAG/OO-2014. The cervical region of the bird was stretched with the necessary force to dislocate the first vertebra attached to the skull. The small intestine and cecum were dissected, removed, and stored at −20°C for later analysis.

### Growth performance

Growth performance was monitored during the initiation stage, finishing stage, and throughout the fattening. For total body weight gain, all broilers were weighed individually on day 0 and subsequently per week, subtracted the initial weight. The weekly food consumption was evaluated by adding the food consumed during all the days of the week. The feed conversion ratio (FCR) was calculated by dividing the grams of feed consumed for weight gain, during each stage and at the end of the experiment.

### Measurement of weight and length of various organs and gastrointestinal tract

On day 42, 10 broilers per treatment were randomly selected and euthanized, and the entire GIT, liver, gallbladder, and spleen were weighed. The GIT of all broilers was measured longitudinally.

### Blood sampling and determinations of blood parameters

On day 42, two mL were collected from the wing vein for each bird into EDTA (5% ethylenediaminetetraacetic acid) tubes for the determination of hematological parameters. Hematocrit determination was performed by the micro-method [13]. Hemoglobin was determined in blood using the cyanometahemoglobin method (Spinreact Drabkin reagent kit; Spinreact Mexico, Naucalpan de Juárez, México). Erythrocyte and leukocyte counts were performed manually with a blood dilution of 1:200 into Natt and Herricks-TIC solution [14]. The differential count of lymphocytes, heterophiles, monocytes, eosinophils, and basophils was determined by Diff Quick stain. The H:L (heterophiles-lymphocytes) index was performed by dividing the number of heterophils by the number of lymphocytes [15]. The second sample was taken in tubes with EDTA (3 mL), centrifuged at 2,000 to 3,000 rpm for 15 to 20 minutes and the serum was obtained to determine concentrations of serum glucose, triglycerides, and cholesterol using GOD-POD Spinreact kits.

### Cytokine and immunoglobulin A levels

The serum levels of the anti-inflammatory cytokines interleukin 10 (IL-10) and gamma interferon (IFN-γ), tumor necrosis factor-β (TNF-β), and pro-inflammatory cytokines TNF-β and IL-1β and IgA serum levels were quantified by ELISA kits following the instructions manufacturer at the 42 days. The kits were as follows: chicken IgA ELISA kit (MyBioSource MBS564152; MyBioSource, Inc., San Diego, CA, USA), chicken TNF-β (MBS778237; MyBioSource, USA), chicken IFN-γ (MBS778165; MyBioSource, USA), chicken IL-10 (MBS778166; MyBioSource, USA), chicken IL-1β (MBS2510251; MyBioSource, USA).

### Statistical analyses

Data are presented as mean values with their standard errors. Data were checked for normal distribution with the Kolmogorov–Smirnov test. One-way analysis of variance was used to analyze the results and the differences between different groups were analyzed by Tukey’s multiple comparisons. It was considered at p<0.05 as significant (Statistica 8.0).

## RESULTS

### Growth performance

#### Body weight gain

During the initiation stage (1 to 21 days) there were no statistically significant changes for body weight gain the values are shown in Table 2. In the same way, the body weight gain was always similar between the treatments during the completion stage and throughout the experimentation period. The group with the highest body weight was PRE, followed by the PRO, which was very similar to the GPA, leaving the CON group and the SYM group below 300 grams.

#### Feed intake

In the same way as the weight gain, during the start stage (1 to 21 days) there were no statistically significant changes in food consumption. From the completion stage (beginning on day 21), a statistically significant decrease was found for GPA, compared to CON (p<0.05). Likewise, there was a statistically significant decrease for PRE and SYM to CON (p<0.05). All values are shown in Table 2.

#### Feed conversion ratio

The feed conversion did not show statistically significant changes in any of the stages or the total fattening. In the completion stage, the PRE treatment improved feed conversion by 14% compared to the CON.

### Weight and length of the organs and the gastrointestinal tract

Table 3 shows the results on the weight of the GIT, liver, gallbladder, and spleen. There was a statistically significant difference in GIT weight between the GPA and the PRE (p <0.05). Coinciding with the weight, the length also showed a significant difference in the length of the GIT, from the crop to the cloaca, of the GPA and PRE (p<0.05). Interestingly, the liver showed the same trend since a statistically significant difference was found between GPA and PRE and PRO (p<0.05).

### Hematological and biochemical parameters

Table 4 shows that no statistically significant difference was found in total erythrocytes but decrease by approximately 30% in GPA and increase by 10% in PRE. No significant differences were found between hematocrit and hemoglobin.

On the other hand, in the total leukocyte count, there was a statistically significant increase in GPA compared to CON and PRE and PRO (p<0.05). Moreover, there were statistically significant changes in lymphocytes and heterophiles in GPA with respect to CON and PRO, and SYM (p<0.05). The H:L ratio was very high in GPA compared to PRO and SYM. While, in the count of monocytes, eosinophils, and basophils there were no significant changes.

For the biochemical parameters analyzed in blood, no statistically significant differences were found between treatments for glucose, cholesterol, or triglycerides (data not shown).

### Cytokine and IgA levels

Table 5 shows that IL-10 levels in the PRO group are lower compared to the CON and PRE groups (p<0.05). For IFN-γ levels, a statistically significant decrease was demonstrated for the GPA and PRO compared to the other 3 groups, mainly CON (p<0.05). Significant differences were found in IL-1β levels, they were observed to increase in SYN compared to CON and GPA and PRE (p<0.05). The TNF-β levels were similar in all treatment groups; therefore, there was no significant difference between them. Interestingly, it was observed that IgA levels decreased by 30% approximately in GPA while in PRO there was an increase of approximately 20% with respect to CON.

## DISCUSSION

This study aimed to evaluate the effect of a probiotic, prebiotic, and symbiotic encapsulated on productive performance, blood, and immune parameters in broilers, compared to colistin sulfate and zinc bacitracin and diet without additives. Table 2 summarized data on the effect of experimental treatments on the growth performance of broilers. No differences were observed in weight gains with any of the treatments compared to the control group. However, the group that gained the most weight at 49 days was the one fed with Agave fructans, although it was not significant. GPA, prebiotic and symbiotic treatments had a lower consumption compared to the control group. However, the FCR only improved by 14% with prebiotic treatment with respect to control group. According to these results, Yang et al [16] reported that treatment with fructooligosaccharides improved feed conversion by 2% to 6%. The improvement in the productive parameters of broilers treated with prebiotics is attributed mainly to the development of beneficial bacteria in the digestive tract, locally to the stimulation of the gut-associated lymphoid tissue (GALT) and a positive modification of the structure and function of the GIT [17]. The chicken microbiota is mainly composed of bacteria of the phylum Firmicutes and Proteobacteria, followed by Bacteroidetes [17]. Fructooligosaccharides treatment (2.5 g/kg diet) increases *Lactobacillus* and limits the growth of *Escherichia coli* and *C. perfringens* [18]. Conversely, the use of zinc bacitracin is associated with a decrease in *Lactobacillus* species [19], which produce short-chain fatty acids that protect the intestinal environment and increase species of Clostridium [20]. In fact, *Lactobacillus*, *Ruminococcus*, and *Clostridium* genera were associated with improvement in productive parameters [17]. Further, the supplementation with 0.2% mannanoligosaccharides (MOS) in a broiler significantly increased the weight of the duodenum, jejunum, and ileum at 42 days, while the group that was administered with oxytetracycline showed a decrease in weight in the jejunum. In the same study, the length of the duodenum increased significantly in chickens fed MOS, and a decrease in length was shown for the group fed with antibiotics [21]. These results coincide with those demonstrated in the present study, where greater weights and lengths of the GIT of the birds of the PRE group were found, compared to the diet with antibiotics. An increase in the weight and length of the small intestine is associated with an increase in the length of the villi in the duodenum, jejunum, and ileum [22]. Thus, the length, number, and surface of intestinal villi are associated to improve the capacity and absorption of nutrients [23,24].

Moreover, the use of probiotics, mainly *bifidobacteria* and *lactobacillus*, has been shown to improve the health status and quality of meat in chickens infected with a pathogenic strain or subjected to stressors [25,26]. According to Khan et al [27], the response to probiotic supplementation is observed after stressful events, including changes in diet, periods of fasting, and changes in temperature; events that did not occur during the present experiment. According to Mookiah et al [28], records of diseases or pathologies should be mentioned in which symbiotics, or probiotics could have had a positive effect, maintaining, or increasing the weight gain of the broiler chicken. Cheng et al [29] evaluated the growth performance of chickens by administering a symbiotic at a dose of 1.5 g/kg with probiotics (*Bacillus subtilis*, *Bacillus licheniformis*, and *Clostridium butyricum*) and prebiotics (yeast cell wall and xylooligosaccharides), showing that the synergy increased daily weight gain and reduced the conversion rate. According to our results, other authors show that the growth performance in chickens does not increase with the dietary inclusion of probiotics and symbiotics compared to the control group [7]. Probably, as demonstrated in the study by Cheng et al [29] a combination of more than one bacterial strain and a prebiotic is necessary to influence the productive parameters.

On the other hand, hematological parameters are used as indicators of health, and variations in these indicators can reflect bacterial, viral, parasitic, or fungal infections, as well as problems of intoxication, dehydration, clotting, or anemia [30]. In general, it is necessary to determine whether the additive introduced to the diet of broilers will induce the integral immune response. According to the present results, a study with *Agave fourcroydes* with high concentrations of fructans did not cause adverse symptoms or significantly decrease hematological parameters [31]. Al-Saad et al [32] reported that treatment with a prebiotic and antibiotic did not modify the red cell count. In the present study, significant changes were found in the differential leukocyte count for the GPA group, which may indicate inflammatory processes due to damage caused by GPAs, indicating that GPAs have local negative effects on GALT through GPA microbial interactions [32]. Regarding hematocrit and hemoglobin, the results coincide with those published by Gutiérrez-Castro and Corredor-Matus [33], where these values were among the normal parameters for the species, they did not show significant changes between treatments. The results are consistent with those reported by Abdel-Hafeez et al [25] where they treated their broilers with a probiotic at a dose of 0.250 kg/ton and a prebiotic at a dose of 2.0, 1.0, and 0.5 kg/ton in the initiation, growth and finishing phases, and found that the treatment did not modify levels of hemoglobin, erythrocytes, glucose, and total cholesterol. In the same way, Nyamagonda et al [34] found that the addition of probiotics and symbiotics to the diet of broilers had no significant effects on the volume of packed cells and hemoglobin.

The H:L index is recognized that it is a good indicator of acute and chronic stressors [35]. Weimer et al [36] indicate that the increase in the proportions of H:L indicates the chronic stress response of the immune system in chickens. The GPA group sees an increase in the H:L ratio, which could demonstrate alteration in the immune system of chickens fed this additive and even the PRO and SYM treatments could function as immunomodulators.

Moreover, we did not find changes in the levels of biochemical parameters due to any of the treatments. According to this, Faramarzzadeh et al [37] showed that the treatment with Chicory fructans in broilers did not induce any significant effect on the concentration of glucose, triglycerides, and total cholesterol. Mokhtari et al [38] found that the serum glucose of broilers was not significantly affected by symbiotics.

In this study, we observed that the IL-10 and IFN-γ levels in the group administered PRO and GPA were lower. This may be due to the interaction that exists between the probiotic and the cells of GALT, generating inhibitory responses on the production of this interleukin. IL-10 plays an important role in viral and parasitic pathogenesis by suppressing the protective immune response of Th1 cells. IFN-γ expression has been associated with viral, bacterial, and parasitic infections; and it is well established that interferon plays a critical role in infection control and pathogen elimination [39]. There are no significant differences in the level of the TNF-β between the groups, this may be due to the physiological conditions of the animals since they were healthy animals in a pathogen-free environment, therefore, the immune responses of the treatments were not observed. IL-1β is produced by a variety of cells after stimulation, particularly by microbes or microbial products [40]. In this regard, in this study, there was an increase of IL-1β in birds treated with symbiotics. A greater understanding of avian mucosal immunity is important, as it can help to develop strategies to prevent bacterial colonization and eventually infection of the mucosal epithelium. This is relevant for both animal welfare and human health. It is well known that the widespread administration of antibiotics is probably an important factor contributing to changes in the mucosal microbiota. There are numerous studies in which the administration of antibiotics such as ciprofloxacin, clindamycin, vancomycin, and ampicillin, among others, generate large long-term changes in the resident microbiota [41]. In this study, we observed that IgA levels tend to decrease in the GPA group, suggesting that dysbiosis alters the GALT mucosa and, consequently, IgA secretion. Likewise, we observed that in the PRO group, the levels tended to increase.

## CONCLUSION

The treatment of prebiotics, symbiotics, and GPA significantly reduced the daily food intake compared to the control group, demonstrating that prebiotics and symbiotics achieve similar productive effects as GPAs. However, prebiotics increases the weight and length of the GIT significantly compared to the group treated with GPA, demonstrating a better development with GIT with prebiotics than with GPA. Probiotic and symbiotic treatments decrease the heterophile-lymphocyte ratio and modify the levels of IL-10, IFN-γ, and IL-1β, suggesting that they may act as immunomodulators. These results suggest that Agave fructans is a novel additive that can be included in the diet of broilers to improve health parameters. However, more studies are suggested with different levels of treatment and also with different probiotic-prebiotic combinations to observe more benefits in broilers.

## Figures and Tables

**Table 1:** Ingredients and composition of the basal diets

Item	Diet^1)^	

	Starter	Grower
Ingredients (%)		
Sorghum	54.47	59.25
Soybean paste	22.5	20.75
Corn gluten meal	7	8
Fish meal	6.16	3
Vegetable oil	6	5.7
Limestome	1.2	1.3
Dicalcium phosphate	1.72	1.22
DL-Methionine	0.2	0
Vitamin-Mineral premix^2)^	0.5	0.5
L-Lysine	0	0.03
Salt	0.25	0.25
Total	100	100
Calculated composition		
Calcium (%)	0.9	0.9
Available phosphorus (%)	0.4	0.35
Arginine (%)	1.3	1.3
Lysine (%)	1.14	1
Methionine (%)	0.53	0.4

1) Starter diet: first 3 weeks; CP, 22.4%; ME, 3.1 kcal/kg. Grower diet: the last 4 weeks; CP, 20.3%; ME, 3.05 kcal/kg.

2) Vitamin A (10,000 IU): Vitamin D_3_ (cholecalciferol), 3,000 IU; Vitamin E (all-rac-α-tocopherolacetate), 30 IU; menadione, 1.3 mg; thiamine 2.2 mg; riboflavin, 8 mg; nicotinamide, 40 mg; choline chloride, 600 mg; calcium pantothenate, 10 mg; pyridoxine·HCl, 4 mg; biotin, 0.04 mg; folic acid, 1 mg; vitamin B_12_ (cobalamine), 0.013 mg; Fe (from ferrous sulfate), 80 mg; Cu (from copper sulfate), 8 mg; Mn (from manganese sulfate), 110 mg; Zn (Bacitracin Zn), 65 mg; iodine (from calcium iodate), 1.1 mg; Se (from sodium selenite), 0.3 mg.

**Table 2:** Effect of the inclusion of encapsulated additives on the growth performance of broilers

Group	CON (mean±SE)	GPA (mean±SE)	PRE (mean±SE)	PRO (mean±SE)	SYM (mean±SE)
1 to 21 days					
BWC (g/bird)	759±16.1^a^	764.6±15^a^	766.4±15.7^a^	776.4±19.2^a^	754.1±17.5^a^
Feed intake (g/bird)	1,210±7^a^	1,145±13^a^	1,182±9^a^	1,165±23^a^	1,167±20^a^
FCR	1.58±0.02^a^	1.49±0.01^a^	1.54±0.04^a^	1.5±0.02a	1.54±0.03^a^
21 to 49 days					
BWC (g/bird)	2,176±118^a^	2,275±96^a^	2,447±98^a^	2,245±114^a^	2,168±86^a^
Feed intake (g/bird)	5,854±29^a^	5594±31^b^	5,612±43^b^	5,691±33^ab^	5,643±61^b^
FCR	2.84±0.2^a^	2.49±0.1^a^	2.32±0.1^a^	2.53±0.03^a^	2.6±0.09^a^
1 to 49 days					
BWC (g/bird)	2,924±113^a^	3,039±87^a^	3,219±90^a^	3,037±110^a^	2,936±81^a^
Feed intake (g/bird)	7,064±49^a^	6,740±20^b^	6,795±14^b^	6,857±11^ab^	6,810±12^b^
FCR	2.4±0.13^a^	2.2±0.1^a^	2.1±0.06^a^	2.2±0.01^a^	2.3±0.07^a^

CON, control; GPA, with growth-promoting antibiotic; PRE, with agave fructan capsules; PRO, with *Bifidobacterium animalis* capsules; SYM, with symbiotic capsules; SE, standard error; BWC, body weight gain; FCR, feed conversion ratio; g, grams.

a,b Different letters indicate significant differences between groups (p<0.05).

**Table 3:** Relative weight and length of organs and gastrointestinal tract of broilers feeding with different additives encapsulated at the end of the experiment

Group	CON (mean±SE)	GPA (mean±SE)	PRE (mean±SE)	PRO (mean±SE)	SYM (mean±SE)
GIT (g)	166.8±6.1^ab^	165.8±4.3^a^	190.3±6.8^b^	184.7±5.7^ab^	175.1±6.6^ab^
GIT (cm)	207±2.4^ab^	186.8±5.6^a^	216.2±6.3^b^	202.8±1.9^ab^	206.4±8^ab^
Liver (g)	53.4±2.7^ab^	47.8±3.4^a^	62.7±1.7^b^	61.8±4.5^b^	51.7±2.4^ab^
Gallbladder (g)	2.3±0.2^a^	2.3±0.1^a^	2.6±0.2^a^	2.1±0.2^a^	2.8±0.2^a^
Spleen (g)	2.8±0.3^a^	2.8±0.1^a^	3.5±0.4^a^	2.8±0.2^a^	3.0±0.3^a^

CON, control; GPA, with growth-promoting antibiotic; PRE, with agave fructan capsules; PRO, with *Bifidobacterium animalis* capsules; SYM, with symbiotic capsules; SE, standard error; GIT, gastrointestinal tract; cm, centimeter; g, grams.

a,b Different letters indicate significant differences between groups (p<0.05).

**Table 4:** Effect of the inclusion of encapsulated additives on the hematological parameters in birds at the end of the experiment

Group	CON (mean±SE)	GPA (mean±SE)	PRE (mean±SE)	PRO (mean±SE)	SYM (mean±SE)
RBC (×10^6^/μL)	1.1±0.1^a^	0.8±0.1^a^	1.2±0.2^a^	0.9±0.04^a^	1.1±0.09^a^
HB (g/dL)	14.0±1.9^a^	13.6±1.5^a^	12.8±1.9^a^	12.4±1^a^	13.0±0.8^a^
HCT (%)	32.6±3.1^a^	31.3±1.3^a^	35.2±1.9^a^	37.7±2.1^a^	33.2±0.8^a^
WBC (×10^4^/μL)	2.5±0.2^a^	3.8±0.3^b^	2.4±0.1^a^	2.5±0.13^a^	2.9±0.2^ab^
Lymphocytes (%)	51.2±5.9^a^	25.8±3^b^	42.2±8.6^ab^	57.2±4.5^a^	57.8±6.2^a^
Heterophils (%)	43±5.4^a^	69.8±4^b^	47.6±7.8^ab^	40±4.3^a^	39±5.8^a^
Monocytes (%)	5.4±1.6^a^	2.4±1^a^	7.4±1^a^	2±0.7^a^	2.6±0.8^a^
Eosinophils (%)	0.2±0.2^a^	1.4±0.5^a^	1.2±0.8^a^	0.4±0.2^a^	0.2±0.2^a^
Basophils (%)	0.2±0.2^a^	0.6±0.4^a^	1.6±0.6^a^	0.4±0.2^a^	0.4±0.4^a^
H:L	0.98	2.91	1.7	0.74	0.75

CON, control; GPA, with growth-promoting antibiotic; PRE, with agave fructan capsules; PRO, with *Bifidobacterium animalis* capsules; SYM, with symbiotic capsules; SE, standard error; RBC, red blood cell; HB, hemoglobin; HCT, Hematocrit; WBC, white blood cells; H:L, heterophile-lymphocyte relationship.

a,b Different letters indicate significant differences between groups (p<0.05).

**Table 5:** Anti-inflammatory and pro-inflammatory cytokines and IgA serum levels in broilers with different encapsulated additives at the end of the experiment

Group	CON (mean±SE)	GPA (mean±SE)	PRE (mean±SE)	PRO (mean±SE)	SYM (mean±SE)
Anti-inflammatory					
L-10 (pg/mL)	428.0±12.2^a^	355.4±13.8^ab^	428.5±31.3^a^	312.7±14.5^b^	384.0±14.8^ab^
IFN-γ (pg/mL)	77.1±10^a^	45.3±6.9^b^	68.9±7.9^ab^	50.3±4.4^b^	67.4±6.1^ab^
Pro-inflammatory					
TNF-β (nmoles/mg)	258.3±34.8^a^	175.0±27.1^a^	261.5±14.6^a^	197.2±34.2^a^	242.4±30.0^a^
IL-1β (pg/mL)	17.6±1.9^a^	18.0±0.5^a^	14.2±1.4^a^	19.6±2.5^ab^	29.1±2.5^b^
IgA (ng/mL)	38.5±4.4^a^	27.3±5.4^a^	38.2±4.1^a^	46.8±9.1^a^	39.1±1.7^a^

CON, control; GPA, with growth-promoting antibiotic; PRE, with agave fructan capsules; PRO, with *Bifidobacterium animalis* capsules; SYM, with symbiotic capsules; SE, standard error; IL-10, interleukin 10; IFN-γ, gamma interferon; TNF-β, tumor necrosis factor beta; IL-1β, interleukin 1 beta; IgA, immunoglobulin A; ng, nanogram; Ml, milliliter; pg, picogram; mg, milligram; nmoles, nanomoles.

a,b Different letters indicate significant differences between groups (p<0.05).

## References

[1] Mehdi Y, Létourneau-Montminy MP, Gaucher ML (2018). Use of antibiotics in broiler production: Global impacts and alternatives. Anim Nutr.

[2] Park SH, Lee SI, Kim SA, Christensen K, Ricke SC (2017). Comparison of antibiotic supplementation versus a yeast-based prebiotic on the cecal microbiome of commercial broilers. PLoS One.

[3] Oh S, Lillehoj HS, Lee Y, Bravo D, Lillehoj EP (2019). Dietary antibiotic growth promoters down-regulate intestinal inflammatory cytokine expression in chickens challenged with lps or co-infected with eimeria maxima and clostridium perfringens. Front Vet Sci.

[4] Terada T, Nii T, Isobe N, Yoshimura Y (2020). Effect of antibiotic treatment on microbial composition and expression of antimicrobial peptides and cytokines in the chick cecum. Poult Sci.

[5] Cardinal MK, Kipper M, Andretta I, Machado Leal Ribeiro A (2019). Withdrawal of antibiotic growth promoters from broiler diets: performance indexes and economic impact. Poult Sci.

[6] Maldonado Galdeano C, Cazorla SI, Lemme Dumit JM, Vélez E, Perdigón G (2019). Beneficial effects of probiotic consumption on the immune system. Ann Nutr Metab.

[7] Sarangi NR, Babu LK, Kumar A, Pradhan CR, Pati PK, Mishra JP (2016). Effect of dietary supplementation of prebiotic, probiotic, and synbiotic on growth performance and carcass characteristics of broiler chickens. Vet World.

[8] Bednarczyk M, Stadnicka K, Kozłowska I (2016). Influence of different prebiotics and mode of their administration on broiler chicken performance. Animal.

[9] Markowiak P, Śliżewska K (2017). Effects of probiotics, prebiotics, and synbiotics on human health. Nutrients.

[10] Juárez-Trujillo N, Jiménez-Fernández M, Franco-Robles E, Beristain-Guevara CI, Chacón-López MA, Ortiz-Basurto RI (2021). Effect of three-stage encapsulation on survival of emulsified Bifidobacterium animalis subsp. Lactis during processing, storage and simulated gastrointestinal tests. LWT.

[11] World Organization for Animal Health (c2018). Animal welfare and broiler production systems [Internet]. Terrestrial Animal Health Code.

[12] Sandoval FJ, Fragoso-Sánchez H (c2014). Broiler welfare manual.

[13] Hashemi R, Majidi A, Motamed H, Amini A, Najari F, Tabatabaey A (2015). Erythrocyte sedimentation rate measurement using as a rapid alternative to the westergren method. Emerg (Tehran).

[14] Acevedo LMR, Blas SSM, Fuentes-Mascorro G (2012). Hemogram and morphological characteristics of blood cells in the olive ridley turtle (Lepidochelys olivacea) of Oaxaca, Mexico. Revista Científica.

[15] Gross WB, Siegel HS (1983). Evaluation of the heterophil/lymphocyte ratio as a measure of stress in chickens. Avian Dis.

[16] Yang Y, Iji PA, Choct M (2009). Dietary modulation of gut microflora in broiler chickens: a review of the role of six kinds of alternatives to in-feed antibiotics. World Poult Sci J.

[17] Pourabedin M, Zhao X (2015). Prebiotics and gut microbiota in chickens. FEMS Microbiol Lett.

[18] Kim GB, Seo YM, Kim CH, Paik IK (2011). Effect of dietary prebiotic supplementation on the performance, intestinal microflora, and immune response of broilers. Poult Sci.

[19] Crisol-Martínez E, Stanley D, Geier MS, Hughes RJ, Moore RJ (2017). Understanding the mechanisms of zinc bacitracin and avilamycin on animal production: linking gut microbiota and growth performance in chickens. Appl Microbiol Biotechnol.

[20] Lu J, Hofacre C, Smith F, Lee MD (2008). Effects of feed additives on the development on the ileal bacterial community of the broiler chicken. Animal.

[21] Alyileili SR, El-Tarabily KA, Belal IEH, Ibrahim WH, Sulaiman M, Hussein AS (2020). Effect of trichoderma reesei degraded date pits on antioxidant enzyme activities and biochemical responses of broiler chickens. Front Vet Sci.

[22] Khan I, Zaneb H, Masood S, Yousaf MS, Rehman HF, Rehman H (2017). Effect of Moringa oleifera leaf powder supplementation on growth performance and intestinal morphology in broiler chickens. J Anim Physiol Anim Nutr (Berl).

[23] Izadi H, Arshami J, Golian A, Raji MR (2013). Effects of chicory root powder on growth performance and histomorphometry of jejunum in broiler chicks. Vet Res Forum.

[24] Teng PY, Kim WK (2018). Review: Roles of prebiotics in intestinal ecosystem of broilers. Front Vet Sci.

[25] Abdel-Hafeez HM, Saleh ES, Tawfeek SS, Youssef IM, Abdel-Daim AS (2017). Effects of probiotic, prebiotic, and synbiotic with and without feed restriction on performance, hematological indices and carcass characteristics of broiler chickens. Asian-Australas J Anim Sci.

[26] El-Sharkawy H, Tahoun A, Rizk AM (2020). Evaluation of bifidobacteria and lactobacillus probiotics as alternative therapy for salmonella typhimurium infection in broiler chickens. Animals (Basel).

[27] Khan AZ, Kumbhar S, Liu Y (2018). Dietary supplementation of selenium-enriched probiotics enhances meat quality of broiler chickens (gallus gallus domesticus) raised under high ambient temperature. Biol Trace Elem Res.

[28] Mookiah S, Sieo CC, Ramasamy K, Abdullah N, Ho YW (2014). Effects of dietary prebiotics, probiotic and synbiotics on performance, caecal bacterial populations and caecal fermentation concentrations of broiler chickens. J Sci Food Agric.

[29] Cheng Y, Chen Y, Li X (2017). Effects of synbiotic supplementation on growth performance, carcass characteristics, meat quality and muscular antioxidant capacity and mineral contents in broilers. J Sci Food Agric.

[30] Giusti M, Lacchini R, Farina OH, Rule R (2012). Biochemical and hematological parameters and productivity of rabbits fed low and normo-protein diets. Acta Bioquim Clin Latinoam.

[31] Iser M, Martínez Y, Ni H (2016). The effects of agave fourcroydes powder as a dietary supplement on growth performance, gut morphology, concentration of igg, and hematology parameters in broiler rabbits. Biomed Res Int.

[32] Al-Saad S, Abbod M, Abo Yones A (2014). Effects of some growth promoters on blood hematology and serum composition of broiler chickens. Int J Agric Res.

[33] Gutiérrez-Castro LL, Corredor-Matus JR (2017). Evaluation of blood parameters and immune response in broilers fed with probiotics. Veterinaria y Zootecnia.

[34] Nyamagonda H, Swamy MN, Veena T, Narayana Swamy HD, Jayakumar K (2009). Effect of prebiotic, probiotic and g-probiotic SPL on certain haematological parameters in broiler chickens. Vet World.

[35] Cotter PF (2015). An examination of the utility of heterophil-lymphocyte ratios in assessing stress of caged hens. Poult Sci.

[36] Weimer SL, Wideman RF, Scanes CG, Mauromoustakos A, Christensen KD, Vizzier-Thaxton Y (2020). Broiler stress responses to light intensity, flooring type, and leg weakness as assessed by heterophil-to-lymphocyte ratios, serum corticosterone, infrared thermography, and latency to lie. Poult Sci.

[37] Faramarzzadeh M, Behroozlak M, Samadian F, Vahedi V (2017). Effects of chicory powder and butyric acid combination on performance, carcass traits and some blood parameters in broiler chickens. Iran J Appl Anim Sci.

[38] Mokhtari R, Yazdani AR, Rezaei M, Ghorbani B (2010). The effects of different growth promoters on performance and carcass characteristics of broiler chickens. J Anim Vet Adv.

[39] Kaiser P, Rothwell L, Goodchild M, Bumstead N (2004). The chicken proinflammatory cytokines interleukin-1beta and interleukin-6: differences in gene structure and genetic location compared with their mammalian orthologues. Anim Genet.

[40] Wigley P, Kaiser P (2003). Avian cytokines in health and disease. Braz J Poult Sci J.

[41] Ubeda C, Bucci V, Caballero S (2013). Intestinal microbiota containing Barnesiella species cures vancomycin-resistant Enterococcus faecium colonization. Infect Immun.

